# Enhanced Swine Behavior Detection with YOLOs and a Mixed Efficient Layer Aggregation Network in Real Time

**DOI:** 10.3390/ani14233375

**Published:** 2024-11-23

**Authors:** Ji-hyeon Lee, Yo Han Choi, Han-sung Lee, Hyun Ju Park, Jun Seon Hong, Ji Hwan Lee, Soo Jin Sa, Yong Min Kim, Jo Eun Kim, Yong Dae Jeong, Hyun-chong Cho

**Affiliations:** 1 Interdisciplinary Graduate Program for BIT Medical Convergence, Kangwon National University, Chuncheon 24341, Republic of Korea; ljhxl2621@kangwon.ac.kr (J.-h.L.); nnn2894@kangwon.ac.kr (H.-s.L.); 2 Swine Science Division, National Institute of Animal Science, Rural Development Administration, Cheonan 31000, Republic of Korea; cyh6150@korea.kr (Y.H.C.); 77qk77@naver.com (H.J.P.); gospel0342@korea.kr (J.S.H.); junenet123@naver.com (J.H.L.); soojinsa@korea.kr (S.J.S.); silveraz@korea.kr (Y.M.K.); kjektw@korea.kr (J.E.K.); yongdaejeong@korea.kr (Y.D.J.); 3 Department of Electronics Engineering, Interdisciplinary Graduate Program for BIT Medical Convergence, and Department of Data Science, Kangwon National University, Chuncheon 24341, Republic of Korea

**Keywords:** deep learning, smart farming technologies, YOLOv7 and YOLOv9, mixed-ELAN, precision agriculture

## Abstract

Effective livestock management has become essential owing to an aging workforce and the growth of large-scale farming operations in the agricultural industry. Conventional monitoring methods, primarily reliant on manual observation, are increasingly reaching their limits, necessitating the development of innovative automated solutions. This study developed a system, termed mixed-ELAN, for real-time sow and piglet behavior detection using an extended ELAN architecture with diverse kernel sizes. The standard convolution operations within the ELAN framework were replaced with MixConv using diverse kernel sizes to enhance feature learning capabilities. To ensure high reliability, a performance evaluation of all techniques was conducted using a k-fold cross-validation (k = 3). The proposed architecture was applied to YOLOv7 and YOLOv9, yielding improvements of 1.5% and 2%, with mean average precision scores of 0.805 and 0.796, respectively, compared with the original models. Both models demonstrated significant performance improvements in detecting behaviors critical for piglet growth and survival, such as crushing and lying down, highlighting the effectiveness of the proposed architecture. These advances highlight the potential of AI and computer vision in agriculture, as well as the system’s benefits for improving animal welfare and farm management efficiency. The proposed architecture enhances the real-time monitoring and understanding of livestock behavior, establishing improved benchmarks for smart farming technologies and enabling further innovation in livestock management.

## Simple Summary

Managing livestock, especially on large-scale farms, is becoming more difficult due to a shortage of labor and an aging farming population. Traditionally, farmers monitor animals manually, which can result in missing important behaviors that impact the health and survival of pigs. This study introduces a new system that uses artificial intelligence to detect critical pig behaviors like crushing and lying down in real time. By improving the capabilities of existing AI models (YOLOv7 and YOLOv9), the system can more accurately monitor pig behavior, helping farmers prevent accidents and ensure better animal welfare. This technology will make it easier to manage livestock more efficiently, improving the quality of life of both animals and farmers.

## 1. Introduction

The livestock industry is critical for sustainable food production worldwide. It faces the challenge of not only increasing production, but also ensuring that production is conducted in an ethically responsible manner. Pork, in particular, has seen an increase in food preferences and consumption worldwide. Global pork production increased from approximately 78 million tons in 1994 to 120 million tons in 2022, making it a significant part of the global food supply [1]. However, global livestock statistics show that there are several risk factors for continuous food supply.

**Figure 1:**
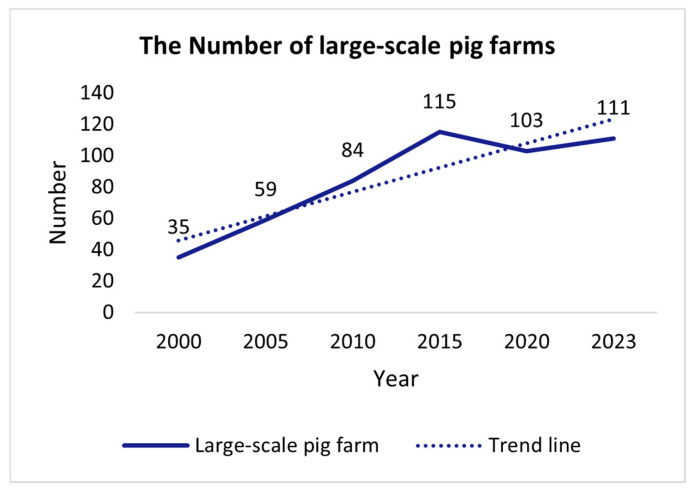
Number of large-scale pig farms in South Korea by year.

First, there has been a demographic shift in livestock farms. The aging rate of livestock farm owners (aged over 65 years) in Korea reached 43.6% in 2019, an increase of 18.4% from 2005. Despite the labor shortage and the aging trend, large-scale farms with more than 10,000 animals have seen an annual growth rate of 7.9%. This makes livestock management increasingly difficult [2]. The annual number of large-scale pig farms in South Korea is shown in Figure 1. This situation is not limited to South Korea. In the EU, for example, the aging rate (age over 65 years) in the livestock industry is 41.3%, and in the United States, the average age of farm owners is 57.5 years. This trend limits the smooth transfer of knowledge and skills to younger generations, which could be detrimental to the sustainability and growth of the livestock industry [3,4,5].

Second, there are limitations in conventional livestock monitoring systems that rely on human resources. When relying on a farmer’s manual observations for farm management, accurately monitoring the nutritional status and activity levels of sows and piglets becomes challenging. For instance, sows nearing farrowing often stand for extended periods, and piglets stressed by cold may show increased overlapping behavior. Additionally, pigs exhibit diverse behaviors based on environmental conditions: Under normal circumstances, they engage in daily behaviors such as eating, drinking, and excreting. Sows display maternal behaviors like nesting and nursing. However, when pigs experience discomfort or illness, they may exhibit abnormal behaviors, including lethargy, aggression, and tail biting. Detecting these behavior changes promptly is essential, as they are often early indicators of health issues. Such variations in behavior can occur at any time of day, and manual observation alone cannot guarantee continuous, accurate detection. Therefore, developing automated monitoring systems powered by AI is crucial to supplement or replace human labor, ensuring precise and timely livestock management [6,7,8].

Therefore, smart farms that integrate internet of things (IoT) and artificial intelligence (AI) into livestock farming are being actively researched [9,10,11,12]. The advantage of smart farming is the ability to monitor livestock without time or location constraints. Studies on the use of AI in the management and monitoring of livestock have shown promising results.

Seo et al. (2020) performed image preprocessing using contrast-limited adaptive histogram equalization (CLAHE), sharpening, and Gaussian filters to detect pigs in images with occlusions. In addition, they proposed an ensemble method that uses the detection box information from two YOLOv4 (You Only Look Once) models in the post-processing stage to improve detection accuracy. This method improves the detection performance by verifying the generated detection boxes through a two-step process. This technique increased the detection accuracy from 79.93% with the standard YOLOv4 to 94.33% [13].

Lai et al. (2023) proposed an improved version of YOLOv5, termed IO-YOLOv5 (illumination–occlusion YOLOv5), for the improved detection of pigs in images with strong occlusions. They introduced a simple attention receptive field block (SARFB) module to expand the receptive field of the model and assign higher weights to important features. In addition, they replaced the conventional spatial pyramid pooling (SPP) module with the ghost spatial pyramid pooling fast cross stage partial connections (GSPPFC) module, which sequentially inputs the input feature map into three 5 × 5 convolution kernels, followed by fusion with the goal of extracting more information with less computational effort. IO-YOLOv5 achieved a 2.2% higher mean average precision (mAP) than the baseline YOLOv5, resulting in a performance increase of 90.8% [14].

Huang et al. (2022) improved the feature extraction network of DarkNet-53 with YOLOv3. They proposed a high-effect YOLO (HE-YOLO) by combining channel attention mechanisms with space attention mechanisms for the detection of pig behaviors such as standing, sitting, prone, and sidling. HE-YOLO showed a performance improvement of 5.61% over YOLOv3, solving the problem of lower mAP in complex breeding environments exhibited by the existing models [15].

Tu et al. (2022) used YOLOX-S and YOLOv5 to improve the identification of pig behaviors, such as lying, eating, and standing, based on video data. They further improved their approach by incorporating a deep simple online and real-time tracking (DeepSORT) algorithm to track identified pigs. This integrated approach substantially improved the multi-object tracking accuracy (MOTA) by 1.8%, reaching 98.6%. This demonstrates the effectiveness of combining object detection and tracking algorithms to achieve better behavior recognition and tracking performance [16].

Gan et al. (2022) conducted a study that focused on detecting the specific behavior of piglets, rather than sows, known as nursing behavior. Instead of training based on a predefined anchor box size, they used an anchor-free deep learning network that identified the object of the centroid and then determined the size and shape of the object to generate anchor boxes accordingly. The predicted anchor boxes were used to compare the intersection over union (IoU) between sows and piglets to detect nursing behavior based on the degree of overlap. The extracted motion features were used to classify the behavior into suckling and non-suckling using a support vector machine (SVM) classifier. Using a dataset of 1 min video clips, their model achieved a 93.6% F1-score, 92.1% recall, and 95.2% precision for detecting nursing behavior [17].

Compared with related studies, the main contributions of this study are as follows: First, the objective was to detect the behavior of both sows and piglets. While most related studies focus solely on the detection of the individual behavior of piglets or sows, this study conducted mutual behavior detection, including crushing and feeding. Second, the detection performance was improved using multiple kernel sizes. In this study, a mixed ELAN (efficient layer aggregation network) using four different kernel sizes was proposed to facilitate effective feature learning and improve the performance of behavior recognition networks. The proposed behavior recognition network is expected to contribute to animal welfare and livestock management automation. Furthermore, it could serve as a basis for increasing livestock production to meet the growing global demand for food.

## 2. Materials and Methods

### 2.1. Dataset

**Figure 2:**
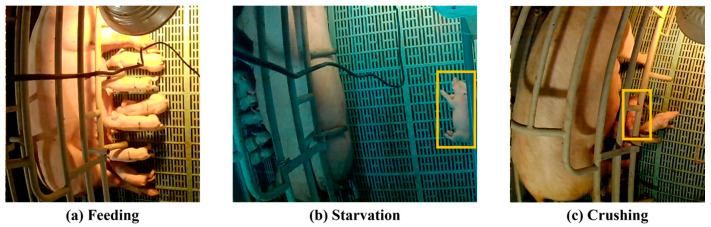
Example images of piglet behaviors.

This study used 1920 × 1080-resolution video data, continuously recorded at 59.89 fps at all hours from 20 August to 18 September 2023 at ProFarm Corporation, an agricultural company in South Korea. The dataset consisted of videos of 50 sows captured using 25 cameras (Fix XA-501). The sows used in this study were two-way crossbreeds of Landrace and Yorkshire, while the piglets were three-way crossbreeds, produced by inseminating these sows with Duroc semen. Each video contained scenes showing the interactions between two sows and various piglet behaviors. The behaviors included four types of sow behaviors (standing, sitting, lying down, lying sideways) and three types of piglet behaviors (feeding, starvation, and crushing). These 5–30 s long videos were extracted frame by frame for training the YOLO network. During this process, the consecutive frames hardly differed in their data characteristics. To create data with diverse features, the frame was extracted at 3 s intervals. This resulted in 2860 images of 50 sows. The data were verified and labeled by livestock experts and divided into training, validation, and test sets in a ratio of 3:1:1 based on sow IDs. Thus, the training set included data collected from 30 sows, whereas the validation and test sets included data collected from 10 sows each. Performance evaluation was conducted using k-fold cross-validation (k = 3), enhancing the robustness of the assessment by mitigating potential overfitting and allowing a more comprehensive evaluation across varied data partitions. This approach ensures that each subset of the dataset contributes to both training and validation, thus strengthening the generalizability of the model’s performance metrics. Table 1 lists the number of labels for each behavior, and Figure 2 shows example images of piglet behaviors, including crushing, feeding, and starvation.

### 2.2. Real-Time Object Detection Model (YOLOv7 and YOLOv9)

Crushing accidents occur in a short period of time, underscoring the need for an algorithm that can promptly identify behaviors in real time. Furthermore, real-time operation is crucial for accurate behavior detection even after the crushing accident has been ruled out. Object detection algorithms have mainly dealt with the YOLO line of models with fast operation speeds. In addition, models such as detection transformer (DETR) have recently been explored to apply transformer architectures to computer vision. Although transformer-based object detection models have a relatively higher detection accuracy than YOLO models, improvements in real-time processing are still needed. Considering the individual characteristics of the model architectures and the necessity for real-time processing, YOLO models were considered suitable. For this study, YOLOv7 and YOLOv9 were used, which are based on the ELAN architecture, and their current state-of-the-art (SOTA) performance was demonstrated.

YOLOv7 improves learning capabilities using expand, shuffle, and merge cardinality operations based on an ELAN architecture that allows layers to learn diverse features through a gradient path [18]. It also applies bag-of-freebies (BoF) techniques to improve performance without increasing the inference cost. Examples of BoF include the proposal of a re-parameterized convolution, RepConvN, and the addition of an auxiliary head to improve performance enhancement through assistant loss.

Wang et al. (2024) highlighted that modern deep learning training methods, such as convolution, transformers, and mamba, overlook losses of information, as the input data are processed through numerous layers [19]. This results in an objective function that does not produce reliable gradients. To solve this problem, YOLOv9 introduced programmable gradient information (PGI) and a generalized efficient layer aggregation network (GELAN). PGI consists of a main branch, an auxiliary reversible branch, and multi-level auxiliary information, which enables the retention of important features even in deep layers without additional costs. The GELAN architecture, which combines ELAN with a cross stage partial network (CSPNet), is an effective layer to improve the complexity, accuracy, and inference speed of the deep learning model. This architecture enables more efficient training than the simple stacking of convolutional layers.

### 2.3. Proposed Layer Block: Mixed-ELAN

Convolutional neural network (CNN) models, including YOLOv7 and YOLOv9, primarily use a kernel size of 3 × 3 for convolution operations. The 3 × 3 convolutions can effectively extract features with fewer parameters and allow the insertion of activation functions between convolutions, increasing the nonlinearity of the model. This theory has been applied since the introduction of visual geometry group (VGG) networks [20].

**Figure 3:**
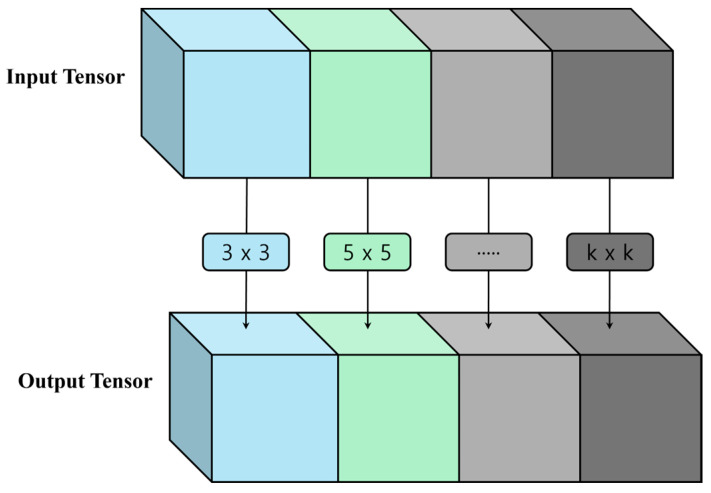
MixConv architecture.

However, several recent studies have reconsidered the concept of optimal kernel size [21,22,23,24]. One of these studies by Liu Z. et al. (2022) argued the limitations of using only one kernel size and proposed an architecture, termed MixConv, that uses both large and small kernel sizes to extract high- and low-resolution features. In general, the parallel application of multiple kernel sizes significantly increases computational complexity. To solve this problem, MixConv uses a group convolution, where the feature map is divided into several groups and different kernel sizes are applied to each group. Figure 3 shows the MixConv architecture, which was calculated using Equations (1) and (2). Equation (1) represents the process of dividing the feature map channels into g groups, and then performing a convolution for each group. X is the input tensor, Y is the output tensor, k is the kernel size of the tth group, m is the channel multiplier, c is the size of the input channel, and W is the weight of the feature map. The final output tensor is obtained by concatenating the tensors using Equation (2).

(1) $${\hat{Y}}_{x,y,z}^{t}=\sum_{-\frac{k_{t}}{2}\leq i\leq \frac{k_{t}}{2},-\frac{k_{t}}{2}\leq j\leq \frac{k_{t}}{2}}{\hat{X}}_{x+i,y+j,z/m}^{t}\cdot {\hat{W}}_{i,j,z}^{t},\;\forall_{z}=1,\ldots ,m\cdot c_{t}$$

(2) $$Y_{x,y,z}=Concat({\hat{Y}}_{x,y,z_{1}}^{1},\ldots ,{\hat{Y}}_{x,y,z_{g}}^{g})$$

**Figure 4:**
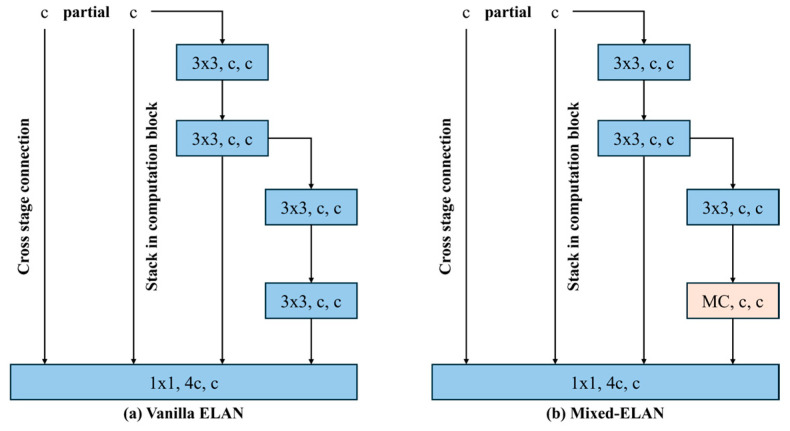
Architecture of vanilla ELAN (**left**) and mixed-ELAN (**right**).

**Figure 5:**
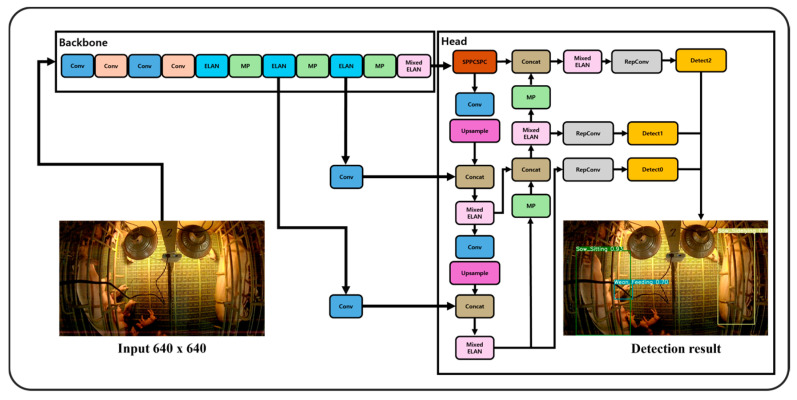
Flowchart of the proposed behavior detection system.

In this study, the tensor operations were performed by dividing them into four groups with kernel sizes of three, five, seven, and nine for each group. These kernel sizes were referenced from those used in Liu Z. et al. (2022) for MixConv. Figure 4 compares the layer structures of vanilla ELAN and mixed-ELAN. Figure 5 shows a flowchart of the proposed behavior detection system.

### 2.4. Evaluation Metrics

To evaluate the performance of the proposed architecture, a commonly used metric for detection models was used, namely, mean average precision (mAP). AP measures the area under the precision–recall curve and reflects the trade-off between precision and recall when the IoU threshold varies. The average AP across all classes is referred to as mAP. Precision refers to the proportion of detected objects that match the ground truth objects, whereas recall refers to the proportion of ground truth objects that the model successfully detects. In this study, an IoU of 0.5 was used as the evaluation criterion.

In addition to mAP, precision and recall were also utilized to further evaluate the model’s detection performance. Precision quantifies the ratio of correctly identified objects to the total objects detected by the model, providing insight into the accuracy of positive predictions. Recall, on the other hand, indicates the proportion of actual objects in the dataset that the model correctly identifies, thereby measuring the model’s ability to detect relevant instances. Together, these metrics offer a comprehensive assessment of the model’s accuracy and sensitivity in detecting pig behaviors under various conditions.

## 3. Results and Discussion

### 3.1. Experimental Setup

The model training was conducted on an NVIDIA TITAN Xp GPU with 12 GB memory, running PyTorch version 2.0.1. For training both YOLOv7 and YOLOv9, we utilized the hyperparameters provided in the ‘hyp.scratch.p5.yaml’ configuration available on GitHub. All training sessions were set to run for 30 epochs, employing the SGD optimizer with a learning rate of 0.01. Additionally, all YOLO models used in the experiments were initialized from pre-trained weights.

### 3.2. Results of YOLOv7 with Mixed-ELAN

Table 2 lists the performance of the original YOLOv7 and YOLOv7 models with mixed-ELAN using k-fold cross-validation (k = 3). The mAP values were above 0.900 for three sow behaviors (standing, sitting, and lying sideways) and relatively low (0.604) for the lying down position. Among the piglet behaviors, the highest mAP (0.764) corresponded to feeding, which has distinct morphological features, followed by crushing (0.645) and starvation (0.629). Because the bounding boxes for piglet behaviors are comparatively smaller than those for sow behaviors, the model must learn to perceive and identify small changes. Consequently, the accuracy in identifying piglet behaviors tends to be lower than that for sow behaviors.

**Figure 6:**
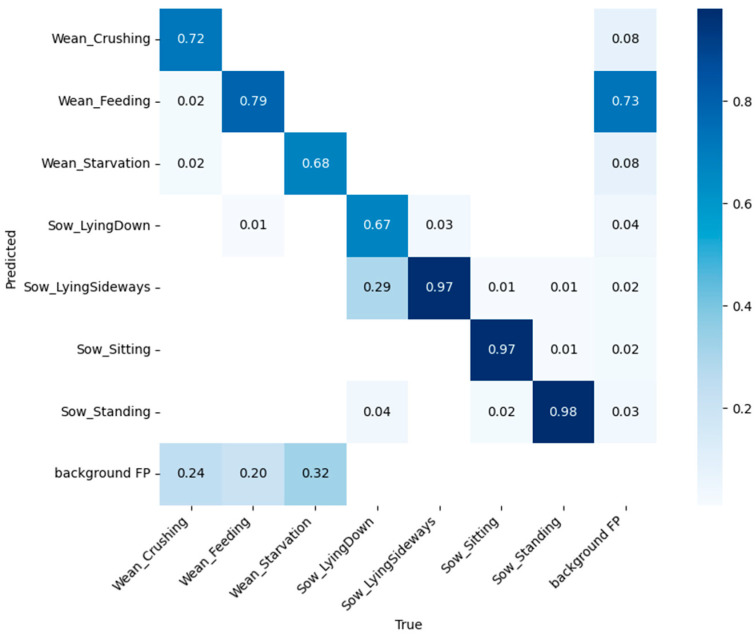
Confusion matrix of YOLOv7 with mixed-ELAN.

YOLOv7 with mixed-ELAN showed an improvement of 5.3% in crushing detection and a 6.3% improvement in lying down detection compared with the YOLOv7 model. Additionally, for crushing detection, precision increased by 5.7% and recall by 1.7%. In pig farming, crushing is the most important behavior to detect as it is linked to piglet survival [25]. In addition, prolonged lying down behaviors of sows can lead to longer fasting times for piglets, which negatively impacts their growth. From this perspective, the improvement in performance with the proposed architecture is extremely beneficial. The detection performance improved by 1.5% across all classes. The confusion matrix of YOLOv7 with the application of Mixed-ELAN is presented in Figure 6.

The position of the mixed convolution layer can significantly affect the performance of the object detection model. Therefore, an ablation study was performed to compare the performance depending on the position of the mixed convolution layer within the ELAN architecture. Table 3 lists the results of applying a mixed convolution layer to each layer. Although layers 1, 2, and 3 showed an improvement in performance for some behaviors, the overall mAP decreased owing to a decrease in other behaviors. In layer 1, the detection performance improved by 12.2% for the lying down position, and decreased for all other behaviors; consequently, the overall performance did not improve. By contrast, layer 4 showed improved performance for the behaviors “lying sideways” and “lying down”, and achieved the highest detection performance for piglet behaviors such as “feeding”, “starvation”, and “crushing”. In particular, for the most important behavior, namely, lying down, the performance improved by 6.3%, and by 5.3% in the case of crushing. The baseline YOLOv7 model demonstrates an inference speed of 48.31 fps, whereas the proposed mixed-ELAN model, incorporating mixed convolution at the fourth layer, achieves an inference speed of 46.95 fps. These results indicate that the proposed model is capable of the real-time detection of pig behaviors.

### 3.3. Results of YOLOv9 with Mixed-ELAN

**Figure 7:**
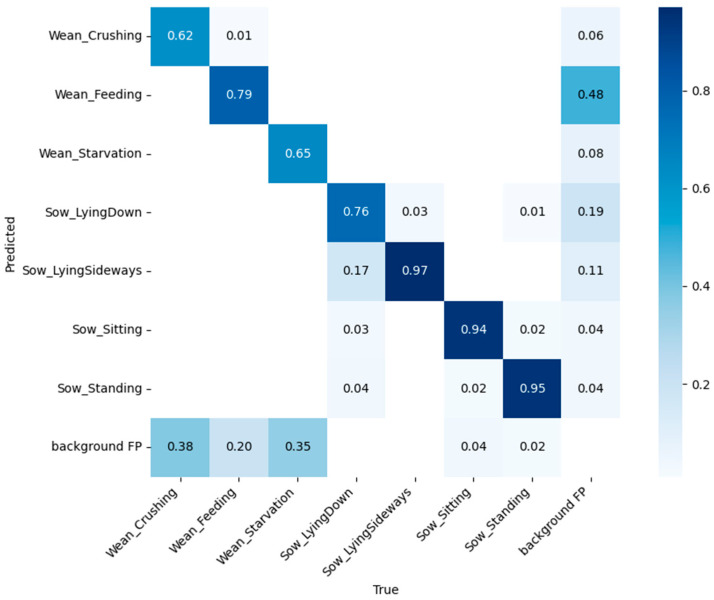
Confusion matrix of YOLOv9 with mixed-ELAN.

Table 4 lists the performance comparison between the original YOLOv9 model and YOLOv9 with mixed-ELAN, while Figure 7 presents the confusion matrix of YOLOv9 with mixed-ELAN. To verify whether the application of mixed convolution has a positive effect on YOLOv7 and YOLOv9, YOLOv9 was trained and evaluated using the results from an ablation study, where mixed convolution was applied to the fourth layer, which was found to yield the best performance. The original YOLOv9 model showed a mAP value of 0.772 for the detection of seven sow and piglet behaviors. Similar to the YOLOv7 results (Table 2), the detection performance for sow behaviors was generally higher than that for piglet behaviors. Among the piglet behaviors, feeding had the highest mAP of 0.772, followed by crushing (0.553) and starvation (0.482). The implementation of mixed-ELAN in YOLOv9 improved the mAP values by approximately 2%. The mAP value improved by 7% to 0.623 for the crushing behavior, and by 7% to 0.554 for the starvation behavior. For crushing detection, the model demonstrated the greatest improvement in precision, with an increase of 10.6%. For the lying down behavior in sows, mixed-ELAN resulted in a 3% improvement in mAP to 0.762, which is similar to the improvement observed with YOLOv7 (Table 2). Similarly, the inference speed of the original YOLOv9 model is 20.08 fps, while the YOLOv9 variant with mixed-ELAN applied achieves 17.30 fps, demonstrating a trend consistent with YOLOv7. This suggests that integrating mixed-ELAN does not significantly compromise the inference speed of the original model.

### 3.4. Discussion

In this study, a mixed convolution was introduced into the ELAN architecture for the improved detection of sow and piglet behaviors. First, an ablation study was conducted for four different positions of the mixed convolution layer within the ELAN architecture to determine the optimal position for the mixed convolution. The results showed that an ELAN architecture with modifications in the last convolution block achieved the highest mAP, which is attributed to the high-dimensional feature information of the deep convolution layers. As the convolution operations progress, the deep learning model generates features that contain multidimensional information extracted by different layers. Performing a mixed convolution on these feature maps with kernels of different sizes helps the model to accurately recognize objects of different sizes and shapes. Moreover, as the modified layer is closer to the final output layer, it effectively extracts features that significantly affect detection. While previous studies have focused on detecting specific behaviors of either piglets or sows individually, such as in [26,27], which addresses specific piglet behaviors, and [28,29,30], which are dedicated solely to sow behavior detection, our study expands this scope by simultaneously detecting critical behaviors of both piglets and sows. This comprehensive approach allows for a more accurate and nuanced understanding of their interconnected behaviors, thus enhancing the robustness of animal welfare monitoring systems.

**Figure 8:**
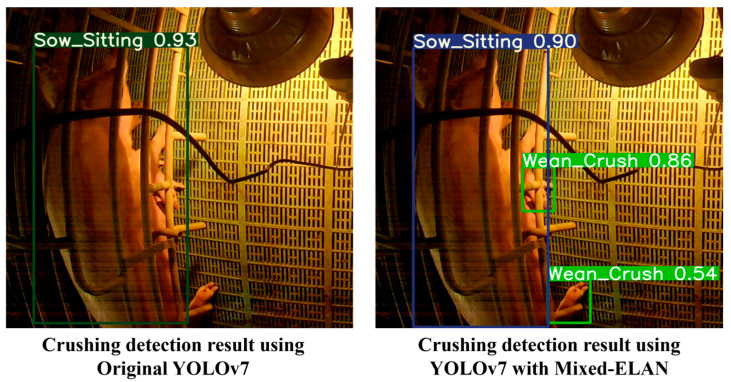
Example of improved detection of crushing behavior in piglets.

The application of mixed-ELAN in both YOLOv7 and YOLOv9 consistently improved the identification of crushing behavior. This indicates that the proposed architecture contributes to the model’s ability to recognize objects through its multikernel operations by comprehensively considering both the object and surrounding environmental information. The proposed architecture improved the detection and identification of crushing and lying down behaviors in piglets and sows, respectively. Figure 8 compares the detection results of YOLOv7 with mixed-ELAN and the original ELAN architecture for crushing behavior in piglets.

While the proposed system demonstrates significant improvements in behavior detection, the practical adoption of AI technologies in livestock farming may face challenges due to initial implementation costs. Many farmers, especially those managing small-to-medium-sized farms, may perceive these systems as an additional financial burden. However, the long-term benefits, including enhanced animal welfare, improved operational efficiency, and reduced labor costs, can outweigh these initial investments. To address these concerns, scalable and modular solutions tailored to the specific needs of farms should be developed. Furthermore, government subsidies or cooperative funding models could play a crucial role in promoting the adoption of AI-driven systems in precision livestock farming.

Furthermore, operational limitations, particularly the system’s reliance on staff for real-time interventions, have been identified, and are addressed as follows: While the system excels in detecting critical behaviors such as piglet crushing, its utility is restricted to time periods when personnel are present. This limitation could be addressed through the integration of automated response mechanisms, such as robotic interventions or real-time alert systems, enabling timely actions even during non-working hours. Additionally, predictive algorithms that analyze historical data could help anticipate high-risk periods, optimizing resource allocation and further enhancing the system’s practicality.

## 4. Conclusions

The objective of this study was to develop a real-time monitoring system to identify sow and piglet behaviors. The YOLOv7 and YOLOv9 models were used to recognize four behaviors of sows and three of piglets. In addition, a modified ELAN architecture, termed mixed-ELAN, was proposed to improve the detection and recognition performance. Mixed-ELAN effectively detects objects of different sizes through convolution operations using kernels of various sizes to extract feature maps. The YOLOv7-based mixed-ELAN achieved a mAP of 0.805 for the seven behaviors, with notable improvements of 6% and 5% in the detection accuracy of lying down and crushing behaviors, respectively. Applying the mixed-ELAN architecture to YOLOv9 improved the mAP values by 2%, and specifically by 7% and 3% for crushing and lying down behaviors, respectively.

Future studies will consider additional methods to improve the accuracy of pig behavior detection. Techniques such as the Convolutional Block Attention Module (CBAM) and Squeeze-and-Excitation Block (SE Block) enhance feature refinement by analyzing the importance of channel and spatial information without substantially increasing computational complexity [31]. Additionally, improving the Feature Pyramid Network (FPN) architecture may better support the detection of piglet behaviors, as FPN leverages multi-scale feature maps to enhance small-object detection. Incorporating these methods into YOLOv7, YOLOv9, and DEtection TRansformers (DETR) could optimize the balance between accuracy and inference speed for more effective behavior recognition in pigs.

## Figures and Tables

**Table 1:** Number of behavior labels in the dataset.

Class		Number of Labels		
		Train	Validation	Test
Sow		3110	1037	1417
	Standing	486	106	330
	Sitting	271	100	154
	Lying down	358	120	141
	Lying sideways	1995	711	792
Piglet		3827	1193	1406
	Feeding	2942	1060	1035
	Starvation	477	107	239
	Crushing	408	26	132
All		6937	2230	2823

**Table 2:** Test results of YOLOv7 and the proposed architecture.

Class		YOLOv7			Mixed-ELAN		
		Precision	Recall	mAP	Precision	Recall	mAP
Sow							
	Standing	0.947	0.942	0.987	0.961	0.909	0.976
	Sitting	0.732	0.972	0.945	0.762	0.925	0.917
	Lying down	0.542	0.742	0.604	0.491	0.778	0.667
	Lying sideways	0.894	0.915	0.961	0.910	0.876	0.972
Piglet							
	Feeding	0.762	0.664	0.764	0.735	0.747	0.782
	Starvation	0.687	0.622	0.629	0.674	0.595	0.622
	Crushing	0.637	0.660	0.645	0.694	0.677	0.698
All		0.743	0.788	0.790	0.747	0.787	0.805

**Table 3:** Ablation study for the optimal position of MixConv in ELAN.

		mAP				
Class		Original	Layer1	Layer2	Layer3	Layer4
Sow						
	Standing	0.987	0.989	0.972	0.978	0.976
	Sitting	0.945	0.904	0.932	0.906	0.917
	Lying down	0.604	0.726	0.720	0.668	0.667
	Lying sideways	0.961	0.964	0.980	0.973	0.972
Piglet						
	Feeding	0.764	0.753	0.762	0.757	0.782
	Starvation	0.629	0.590	0.518	0.609	0.622
	Crushing	0.645	0.602	0.635	0.597	0.698
All		0.790	0.790	0.788	0.784	0.805

**Table 4:** Test results of YOLOv9 and the proposed architecture.

Class		YOLOv9			Mixed-ELAN		
		Precision	Recall	mAP	Precision	Recall	mAP
Sow							
	Standing	0.934	0.946	0.979	0.926	0.907	0.962
	Sitting	0.699	0.957	0.909	0.760	0.954	0.941
	Lying down	0.601	0.831	0.735	0.644	0.747	0.762
	Lying sideways	0.888	0.952	0.969	0.906	0.958	0.965
Piglet							
	Feeding	0.726	0.729	0.772	0.738	0.701	0.765
	Starvation	0.608	0.476	0.482	0.648	0.493	0.554
	Crushing	0.507	0.650	0.553	0.613	0.626	0.623
All		0.709	0.792	0.772	0.748	0.770	0.796

## Data Availability

The raw data supporting the conclusions of this article will be made available by the authors on request.
